# Corrosion and Interfacial Contact Resistance of NiTi Alloy as a Promising Bipolar Plate for PEMFC

**DOI:** 10.3390/molecules29153696

**Published:** 2024-08-05

**Authors:** Yingping Li, Xiaofen Wang, Yuanyuan Li, Zhuo He, Guohong Zhang, Zhen Wang, Shaohua Wang, Fei Hu, Qiongyu Zhou

**Affiliations:** 1 The State Key Laboratory of Refractories and Metallurgy, Wuhan University of Science and Technology, Wuhan 430081, China; liyingping@wust.edu.cn; 2 Key Laboratory of Green Surface Technology and Functional Coatings for Materials, China National Light Industry, Foshan University, Foshan 528000, China; wangxiaofen@shu.edu.cn (X.W.); 20220580212@stu.fosu.edu.cn (Z.H.); mfhufei@126.com (F.H.); zhouzhouqiongyu@fosu.edu.cn (Q.Z.); 3 School of Materials Science and Engineering, Shanghai University, Shanghai 200444, China; 4 Analytical and Testing Center, Wuhan University of Science and Technology, Wuhan 430081, China; zhangguohong@wust.edu.cn (G.Z.); wangzhen@wust.edu.cn (Z.W.); 5 School of Materials Science and Engineering, Jingdezhen Ceramic University, Jingdezhen 333403, China

**Keywords:** NiTi alloy, corrosion resistance, interfacial contact resistance, bipolar plates, PEMFC

## Abstract

Titanium (Ti) is generally considered as an ideal bipolar plate (BPP) material because of its excellent corrosion resistance, good machinability and lightweight nature. However, the easy-passivation property, which leads to increased interfacial contact resistance (ICR) and subsequently decreased cell performance, limits its large-scale commercial application in proton exchange membrane fuel cells (PEMFCs). In this paper, we proposed a NiTi alloy prepared by suction casting as a promising bipolar plate for PEMFCs. This NiTi alloy exhibits significantly decreased ICR values (16.8 mΩ cm^2^ at 1.4 MPa) compared with pure Ti (88.6 mΩ cm^2^ at 1.4 MPa), along with enhanced corrosion resistance compared with pure nickel (Ni). The superior corrosion resistance of NiTi alloy is accredited to the nobler open circuit potential and corrosion potential, coupled with low corrosion current densities and passive current densities. The improved ICR can be interpreted by the existence of high-proportioned metallic Ni in the passive film, which contributes to the reduced capacitance characteristic of the passive film (compared with Ti) and enhances charge conduction. This work provides a feasible option to ameliorate BPP material that may have desirable corrosion resistance and ICR.

## 1. Introduction

To alleviate the increasingly severe energy shortage and environmental issues caused by the continuous consumption of non-renewable fossil fuels, the development of clean and efficient energy sources has attracted significant attention [1,2]. Among these sources, hydrogen is considered an ideal environmentally friendly alternative to conventional fuels due to its high calorific value and negligible environmental pollution [3,4]. The efficient utilization of hydrogen energy necessitates the advancement of innovative technologies, with the proton exchange membrane fuel cell (PEMFC) being one of the most advanced and efficient energy generation methods for converting chemical energy (hydrogen and oxygen gases) into electricity [5,6]. However, the widespread usage of PEMFCs is still hindered by its high-cost components [7]. The membrane electrode assembly (MEA), gas diffusion layer (GDL) and bipolar plates (BPPs) are the main components of PEMFC [8,9,10]. Among these, the bipolar plates are considered as highly critical components, because they account for 80% of the total weight and 40% of the total cost of PEMFCs [11,12]. These plates serve to collect current, as well as separate and distribute reactants and oxidant gases. The acidic working environment of the PEMFC can lead to corrosion damage to BPPs, which in turn reduces the output power and service life of the fuel cell [13]. Therefore, exploring advanced strategies or new materials to develop BPPs with enhanced anti-corrosive performance, high mechanical strength and superior electrical conductivity is highly anticipated [14].

Recently, several materials have shown promise as competitive options for BPPs. Graphite and its composites have the advantages of excellent electrical conductivity and chemical stability, but the poor manufacturability, durability and permeability limit their commercial application [15,16]. Unlike the brittle nature of graphite and its composites, metallic BPPs (such as those made from Ni, aluminum, titanium and stainless steel, etc.) can satisfy the requirements for mechanical properties, gas permeability and manufacturing costs [17]. Among these materials, titanium and its alloys stand out due to their high specific strength and exceptional corrosion resistance in harsh environments [5]. However, titanium and its alloys face challenges related to the increased interfacial contact resistance, attributed to the nonconductive passive film formed on the surface in the service process. Although the passive film can enhance the corrosion resistance, there still exists non-negligible issues in impairing electrical conductivity, leading to power waste and degenerate electric output in PEMFCs [18,19].

In this work, we introduce a NiTi alloy with superior corrosion resistance and favorable ICR, which makes it a promising material for BPPs to be used in PEMFCs. Compared with commercial Ti plate, the NiTi alloy has a fairly improved ICR value of 16.8 mΩ cm^2^ at 1.4 MPa. Additionally, the NiTi alloy shows much better corrosion resistance compared with commercial Ni plates. Furthermore, the surface states of NiTi alloy in simulated PEMFC environments have been investigated to analyze its remarkable performance.

## 2. Results and Discussion

**Figure 1:**
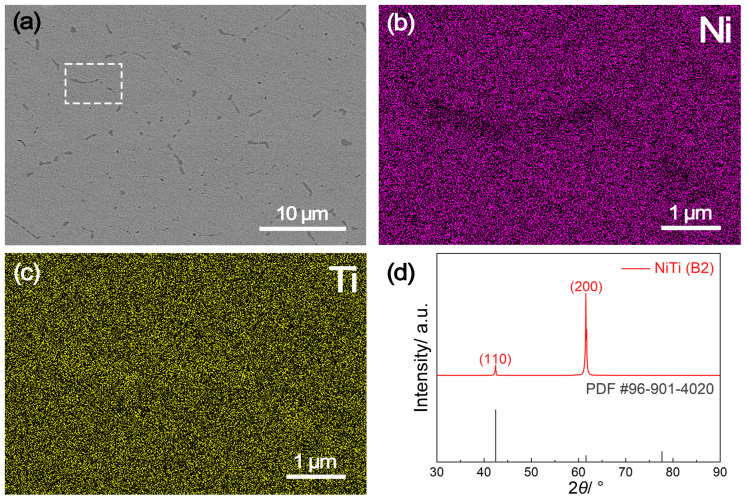
The (**a**) scanning electron microscopy (SEM) image and (**b**,**c**) corresponding elemental mapping results of the indicated region (white square in subfigure (**a**)) of the NiTi alloy prepared by suction casting, along with (**d**) the X-ray diffraction (XRD) pattern.

The microscopic morphology, elemental distribution and phase constituents of the NiTi alloy sample prepared by suction casting are shown in Figure 1. The scanning electron microscopy (SEM) image reveals a homogeneous microstructure with minimal defects (Figure 1a), owning to the rapid solidification process employed during suction casting. The darker regions observed mainly at brain boundaries are identified to contain NiTi_2_ precipitates [20]. According to energy dispersive spectroscopy (EDS) mapping results (Figure 1b,c) from the indicated region in Figure 1a, Ni and Ti elements distribute homogeneously within the grain interior, but show depletion and enrichment at grain boundaries, respectively, because of the presence of NiTi_2_. Analysis of the X-ray diffraction (XRD) pattern (Figure 1d) indicates that the diffraction peak positions of the as prepared NiTi alloy agree well with those of the standard austenitic B2 phase (JCPDS No. 96-901-4020) [21], confirming the consistency in phase structure. However, the shift of the highest diffraction peak from (110) to (200) suggests the presence of strong texture that formed during rapid solidification. Notably, the texture also results in the absence of detectable NiTi_2_ phase in XRD analysis. These characteristics of the NiTi alloy sample prepared by suction casting are quite different from that of the NiTi alloy prepared by conventional melting, which exhibits the existence of as-cast dendrites and a lack of strong texture (as shown in Figure S1, Supplementary Materials).

**Figure 2:**
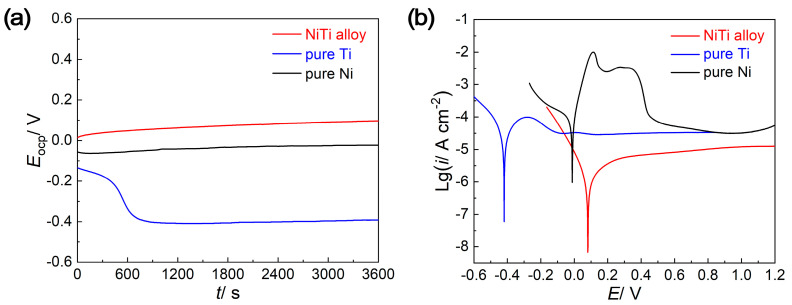
The (**a**) open circuit potential versus time (*E*_OCP_–*t*) curves and (**b**) potentiodynamic polarization curves of NiTi alloy, pure Ti and pure Ni.

Evolution of open circuit potential (*E*_OCP_)with the immersion time and the potentiodynamic polarization results are employed to investigate the corrosion resistance of NiTi alloy, pure Ti and pure Ni in 0.5 mol/L H_2_SO_4_ + 2 ppm HF solutions. As shown in the *E*_OCP_–*t* curves in Figure 2a, the *E*_OCP_ of pure Ti electrode decreases at the initial stage of immersion and then reaches a stationary value, while the *E*_OCP_ of NiTi alloy and Ni increases gradually and then tends to be stable. The two different tendencies at the initial stage of immersion may be caused by the naturally formed passive films on the surface, which is an intrinsic character that would be destroyed in the solution that contains halide ions (specifically F^−^ ions in this work) [22]. The *E*_OCP_ stabilizes after a long immersion for all samples, meaning that the dissolution and growth of the passive films reach a dynamic equilibrium. When compared with pure Ti and pure Ni, the NiTi alloy displays a more positive *E*_OCP_ value, signifying the higher inertness property and expected superior corrosion resistance [23].

The potentiodynamic polarization curves are exhibited in Figure 2b, and the corresponding electrochemical parameters are listed in Table 1. Clearly, the corrosion potential (*E*_corr_) of NiTi alloy is much nobler than that of pure Ti and pure Ni, and the current density (*i*_corr_) of NiTi alloy is an order of magnitude lower than that of pure Ti and pure Ni. In addition, typical passivation behavior can be observed in the anodic branches of potentiodynamic polarization curves for all samples, with the contribution of passive elements Ti and Ni [24]. However, both the anodic branches of pure Ti and pure Ni possess the active–passive dissolution process, which is generally considered to be the oxidation process of the unstable transition oxides (sub-oxides) [25]. In contrast, the anodic branch of NiTi alloy changes directly into the passive region without an active–passive transition, indicating the superior passive property that the protective film can be formed almost spontaneously at the *E*_corr_ [26]. Furthermore, the NiTi alloy has a lower passive current density (*i*_pass_) than that of pure Ti and pure Ni, which can also reveal a better passive property. By comparing the electrochemical parameters related to general corrosion (*E*_ocp_, *E*_corr_, *i*_corr_) and the passivation characteristics of the anodic process (*i*_pass_), it can confirm the superior electrochemical stability of NiTi alloy in the acidic solution containing F^−^ ions.

**Figure 3:**
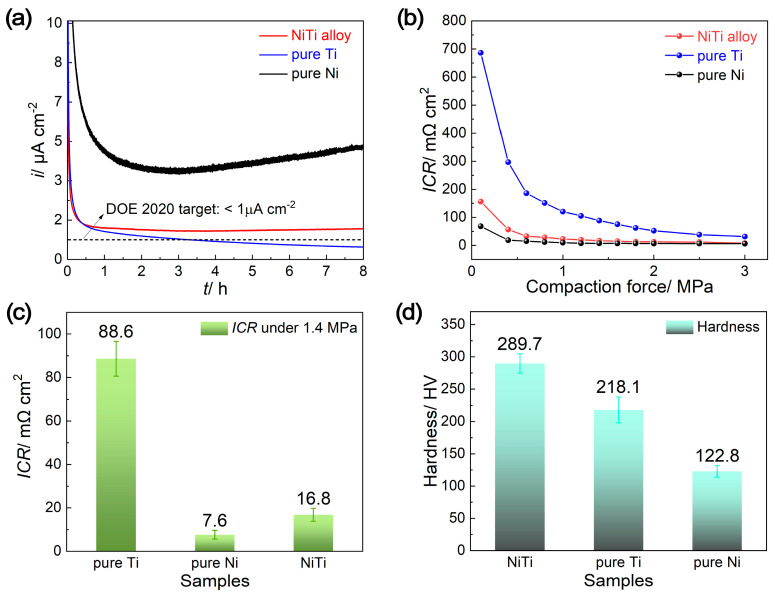
The (**a**) potentiostatic polarization curves, interfacial contact resistance (ICR) values: under (**b**) different compaction force and (**c**) the pressure of 1.4 MPa, along with (**d**) the hardness of NiTi alloy, pure Ti and pure Ni.

The critical properties for BPPs, including durability, interfacial contact resistance (ICR) and hardness, are displayed in Figure 3. Potentiostatic polarization tests at a simulated cathodic working potential (+0.85 V vs. SHE) were carried out to assess the durability requirements in a PEMFC environment (Figure 3a). At the initial stage, the current densities (*i*_cathode_) for all samples decrease rapidly to lower values, because of the formation of passive films at this working potential, which lies within the passive regions for all samples (Figure 2b) [21,22,23,24,25]. Afterwards, pure Ni shows a relatively unstable and higher current density, signifying more electrochemical reactions and dissolution of metal ions from the Ni electrode [27]. For the NiTi alloy plate, its current density is slightly higher than the DOE standard of less than 1 μA cm^−2^, measuring at 1.28 μA cm^−2^. This indicates the good durability of the NiTi alloy, proving it to be a potential material for BPPs. The current density of the pure Ti plate is slightly lower than that of the NiTi alloy plate, confirming its preeminent corrosion resistance, due to the rapid oxidation on its surface to form TiO_2_ in the simulated cathode environment. However, the passive film formed on the surface of the Ti plate could result in an increased ICR value and the degradation of the cell’s performance [24].

The ICR between the BPPs and the GDL, one of the most significant resistances occurring at all interfaces within the PEMFC stack, is another critical factor affecting fuel cell performance. Figure 3b,c show the ICR values of the NiTi alloy, pure Ti and pure Ni after 8 h potentiostatic polarization at a cathode potential of 0.85 V vs. SHE in a 0.5 M H_2_SO_4_ and 2 ppm HF solution, under varying compaction forces and a pressure of 1.4 MPa. As shown in Figure 3b, the ICR values for all samples decrease rapidly at low compaction force, and then decrease slightly after reaching the pressure of 1.4 MPa (a typical compaction force for a single cell), revealing that the higher pressure ensures sufficient contact areas between BPPs and GDL [27]. Under each pressure, the ICR values are in the order of pure Ni < NiTi alloy < pure Ti. The ICR of the NiTi alloy (16.8 mΩ cm^2^) especially, is rather lower than that of pure Ti (88.6 mΩ cm^2^) under the pressure of 1.4 MPa, and is close to that of pure Ni (7.6 mΩ cm^2^) (Figure 3c). The ICR of NiTi alloy is only slightly higher than the US DOE 2020 requirement (10 mΩ cm^2^ under a compaction force of 1.4 MPa), but can be comparable with many surface treatment coatings (e.g., Cr electroplating coatings, double cathode glow discharge ZrCN nanocrystalline coatings, plasma-nitrided layers, etc.) [23,28]. Compared with these surface coatings, the NiTi alloy offers a significant advantage because of its simplified fabricating process and lower fabrication cost.

In addition, the hardness of the NiTi alloy (289.7 HV) is higher than that of pure Ti and pure Ni (218.1 and 122.8 HV, respectively) (Figure 3d), which contributes to enhancing the mechanical strength of the BPPs. Therefore, the NiTi alloy prepared by suction casting, as presented in this study, showcases a significant potential as BPP materials in PEMFCs, due to its considerable corrosion resistance (compared with pure Ni), lower ICR (compared with pure Ti) and superior mechanical strength.

**Figure 4:**
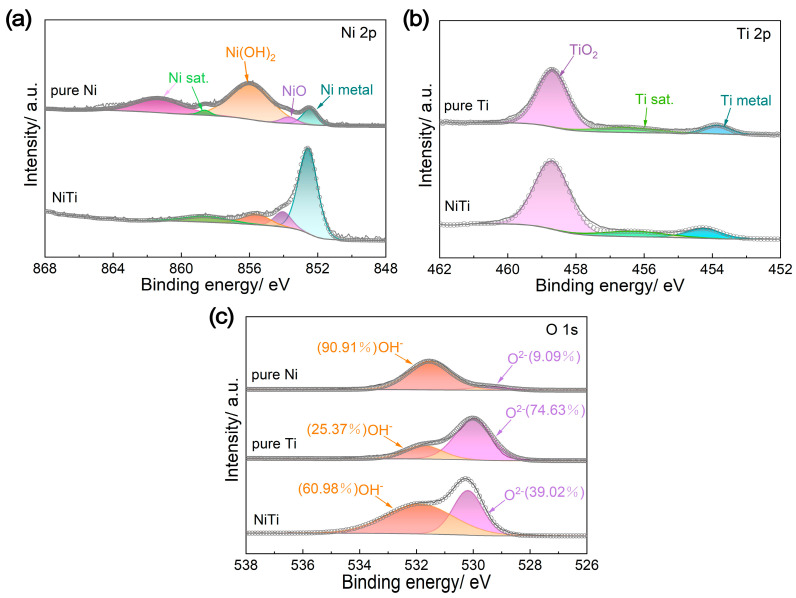
X-ray photoelectron spectroscopy (XPS) analysis results of different materials (NiTi alloy, pure Ti and pure Ni): (**a**) Ni 2p, (**b**) Ti 2p and (**c**) O 1s.

To thoroughly understand the remarkable performance of NiTi alloy in terms of corrosion resistance and ICR, the surface states of NiTi alloy, pure Ti and pure Ni were analyzed by X-ray photoelectron spectroscopy (XPS), electrochemical impedance spectroscopy (EIS) and scanning electron microscopy (SEM). According to the deconvoluted XPS spectra for Ni 2p and Ti 2p (Figure 4a,b), Ni(OH)_2_ (855.6 eV), NiO (853.7 eV) and metallic Ni (852.6 eV) are present on the surfaces of both the NiTi alloy and pure Ni, while TiO_2_ (458.5 eV) and metallic Ti (454.1 eV) are found on the surfaces of both the NiTi alloy and pure Ti [29]. Meanwhile, the O 1s spectra consist of O^2−^ (530.0 eV) and OH^−^ (531.6 eV) peaks (Figure 4c) [30]. The OH^−^ species can originate from hydrated metal oxides or adsorbed H_2_O [26]. The estimated ratios of different chemical states on the surfaces of NiTi alloy, pure Ti and pure Ni are listed in Table 2. When compared with pure Ti, the NiTi alloy has a relatively higher proportion of metallic components (76.34% metallic Ni, 12.28% metallic Ti), which is related to the metallic-type electrical conduction mechanism and contributes to high electrical conductivity and low ICR [29,31]. However, the presence of nonconductive TiO_2_ species on the surface of the NiTi alloy leads to its ICR being slightly higher than that of pure Ni (Figure 3c). The ratio of O^2−^/OH^−^ also reflects the surface condition of the metals, representing the oxide content in the passive film [32]. Apparently, the pure Ti has the highest O^2−^/OH^−^ ratio (2.94), indicating good corrosion resistance but poor electrical conductivity due to the dense passive film. Conversely, pure Ni exhibits the opposite effect, with an O^2−^/OH^−^ ratio of 0.10. Consequently, the NiTi alloy possesses a moderate O^2−^/OH^−^ ratio (0.64), achieving a good balance between anti-corrosion and electrical conductivity performance. Moreover, an appropriate passive film on the surface of NiTi alloy can also prevent Ni ions from leaching [33,34].

**Figure 5:**
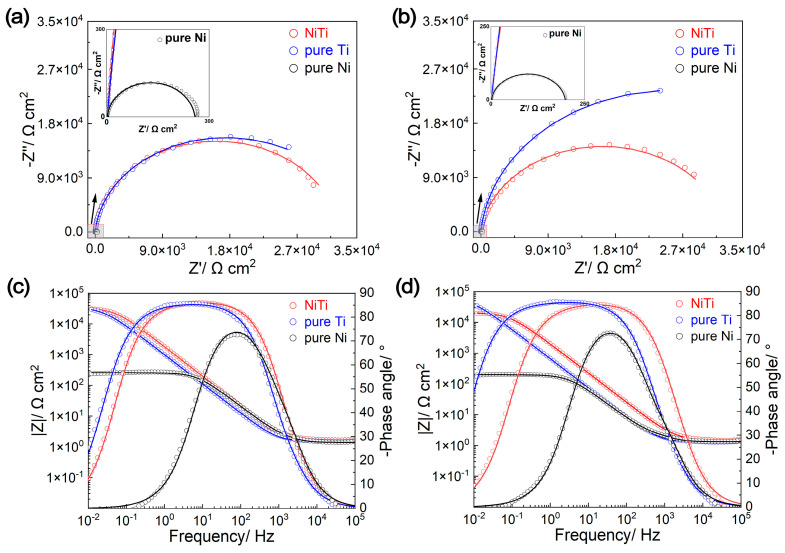
Electrochemical impedance spectroscopy (EIS) analysis of NiTi alloy, pure Ti and pure Ni before ((**a**) Nyqusit plots, (**c**) Bode-phase plots) and after ((**b**) Nyqusit plots, (**d**) Bode-phase plots) potentiostatic polarization.

The EIS results are displayed in Figure 5, and have been fitted to an equivalent circuit (EEC, shown in Figure S2, Supplementary Materials), where *R*_s_ represents the solution resistance, *R*_1_ and *R*_2_ represent the charge transfer resistance and passive film resistance, respectively, and CPE_1_ and CPE_2_ represent the contrast phase elements corresponding to the double-layer capacitance and passive film. The fitting results are shown in Table 3, with chi-square (χ^2^) values in the range of 10^−4^~10^−3^, indicating that the EEC reasonably reflects the actual electrochemical process occurring on the surface of the tested samples in the simulated PEMFC environment. From the Bode-phase angle plots (Figure 5c,d), it can be observed that the log|Z| and log|*f*| of the NiTi alloy and pure Ti exhibit linear relationships over a wide frequency range, with slopes close to −1. However, Ni only shows a linear relationship within a narrow frequency band in the mid-frequency region (*f*: 10^3^~10^1^ Hz), signifying that the corrosion resistance of the NiTi alloy and pure Ti is significantly better than that of Ni [22]. Additionally, both the NiTi alloy and pure Ti display a platform in the mid-to-low-frequency range, with their maximum phase angles approaching 90°, suggesting obvious capacitive characteristics related to the protective passive films [19,23]. In contrast, pure Ni displays a time constant in the mid-frequency region, which is characteristic of metallic material in electrolytes [26,35]. In addition, the capacitive characteristics of pure Ti are stronger than those of the NiTi alloy, which can be concluded from the fact that (1) the *Z*” parts in the Nyqusit plots of pure Ti are larger than those of the NiTi alloy (Figure 5a,b) and (2) *Y*_0–pure Ti_ > *Y*_0–NiTi alloy_ (Table 3). Here, the thickness of passive film (δ) can be compared by 1/Y_0_ [36]. These results can effectively account for the ICR order: pure Ni < NiTi alloy < pure Ti.

**Figure 6:**
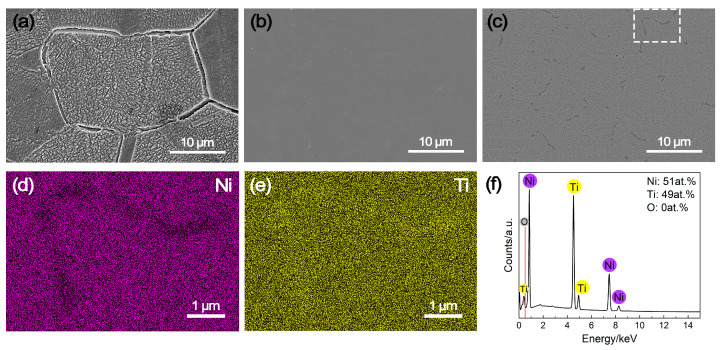
SEM images of (**a**) pure Ni, (**b**) pure Ti and (**c**) NiTi alloy after potentiostatic polarization measurements, along with (**d**,**e**) elemental mapping results and (**f**) semi-quantitative result of the indicated region (white square in subfigure (**c**)).

The SEM images and EDS results of the NiTi alloy, along with pure Ni and pure Ti as comparative samples, following potentiostatic polarization measurements are illustrated in Figure 6. As evident in Figure 6a, pure Ni suffered from severe intergranular corrosion under the cathode operating condition of the PEMFC, leading to the unstable cathodic current densities during the potentiostatic polarization over time (Figure 3). In contrast, pure Ti reveals almost no evidence of corrosion (Figure 6b), which benefits from the passivation process in the meantime. Similarly, there is no sign of corrosion on the NiTi alloy either (Figure 6c). Additionally, the EDS mapping results of the indicated region in Figure 6a differ little from those obtained before potentiostatic polarization. While the grain boundaries of the NiTi alloy display depletion of Ni and enrichment of Ti, the elemental distribution within the grain interior remain uniform (Figure 6d,e). It is noteworthy that the semi-quantitative analysis of NiTi alloy did not detect the presence of oxygen (Figure 6f), implying the formation of a relatively thinner passive film on the NiTi alloy surface. These results can thoroughly confirm the high corrosion resistance of NiTi alloy, which is similar to pure Ti.

## 3. Materials and Methods

Electrolytic Ni (purity > 99.9%) and sponge Ti (purity > 99.7%) with an atomic ratio of 51:49 were utilized to fabricate melting cast NiTi alloy in a non-consumable vacuum arc melting furnace (WK-I, Physcience Opto-electronics Co., Ltd., Beijing, China). Then, the rectangular NiTi plates measuring 20 mm × 10 mm × 1 mm were obtained by suctioning the molten alloy into a water-cooled copper mold. Meanwhile, pure Ti and pure Ni with the same dimensions were also used in this study for comparative analysis. Thereafter, all samples were grinded and polished, as well as cleaned with deionized water and anhydrous ethanol.

The OCP, potentiodynamic polarization, potentiostatic polarization and EIS measurements were conducted to investigate the electrochemical performance of NiTi alloy in the electrolytic cell solution (0.5 mol/L H_2_SO_4_ + 2 ppm HF), which is usually used to simulate the working environment of the fuel cell. All tests were performed using a CHI660E electrochemical workstation (Chenhua, Shanghai, China), employing a three-electrode electrochemical cell, consisting of the NiTi alloy serving as the working electrode, a Pt sheet as the counter electrode, and a saturated Hg/Hg_2_SO_4_ electrode as the reference electrode. In this paper, all experiments were repeated at least three times to ensure accuracy. Furthermore, the obtained potentials (V vs. Hg/Hg_2_SO_4_) were converted to potentials versus a standard hydrogen electrode (SHE) according to the relationship: V vs. SHE = V vs. Hg/Hg_2_SO_4_ + 0.658.

Initially, the OCP versus time relationship was recorded for 3600 s to obtain a stable surface condition. The potentiodynamic polarization was measured in the range of −0.6 V vs. SHE to 1.0 V vs. SHE with a scan rate of 1 mV/s. The potentiostatic polarization was conducted at a potential of 0.85 V vs. SHE for 8 h. The EIS measurement was tested at the OCP frequency range spanning from 100 kHz to 10 mHz, applying a perturbation magnitude of 10 mV.

The microstructure and corresponding elemental distribution were characterized by SEM (Apreo S Hivac, Thermo Fisher, Waltham, MA, USA) equipped with EDS (Ultim Live 100X, Oxford, Abingdon, England). The crystal structure was analyzed by XRD (SmartLab SE, Rigaku, Tokyo, Japan) with Cu Kα radiation at a scanning step 0.02° and a scanning speed of 10°/min. The composition and chemical state of the passivation film formed on the sample surface were analyzed using XPS (AXIS SUPRA+, Shimadzu, Kyoto, Japan). The hardness was tested using a small load Vickers hardness tester (HV-1000B, Huayin, Wuhan, China) with a loading force of 500 g and a holding time of 20 s. Each sample underwent 10 measurements and the average value was taken.

**Figure 7:**
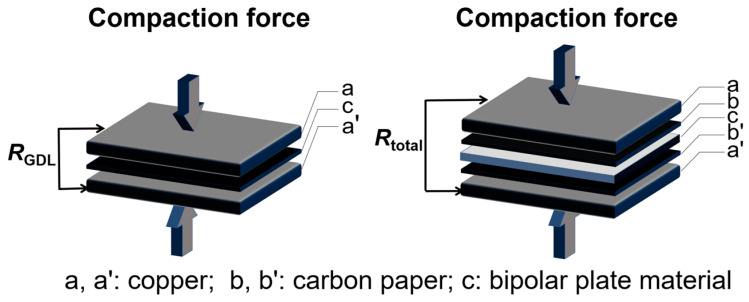
The schematic diagram for testing the *R*_GDL_ and *R*_total_ values.

The ICR values were measured by a sandwich configuration proposed by Wang et al. [27], as shown in the schematic diagram in Figure 7, and calculated using following equation:

$$\;ICR=S(R_{total}-R_{GDL})/2\;$$

where *S* is the contact area between the sample and the carbon paper. *R*_total_ signifies the measured total resistance, whereas *R*_GDL_ denotes the resistance attributed to the GDL within carbon paper. The operating load spanned from 0.1 to 3.0 MPa, containing the range used to record the ICR values under different compaction forces.

## 4. Conclusions

A NiTi alloy bipolar plate material was prepared by suction casting and its corrosion resistance, ICR and hardness were investigated in the simulated PEMFC environments. The NiTi alloy has a fairly low ICR value of 16.8 mΩ cm^2^ at 1.4 MPa, which is a considerable improvement over the pure Ti plate (88.6 mΩ cm^2^ at 1.4 MPa) and slightly higher than the US DOE 2020 target. Additionally, the NiTi alloy shows superior corrosion resistance in electrochemical measurements and remains corrosion-free after potentiostatic polarization in a simulated cathodic environment for 8 h. In addition, the NiTi alloy has a higher hardness (289.7 HV) compared with pure Ti (218.1 HV) and pure Ni (122.8 HV). Overall, this work showcases the significant potential of the NiTi alloy prepared by suction casting as a BBP material in PEMFCs. In the near future, further investigation is warranted on the long-term use of NiTi alloy for BPPs, including factors such as pH value, fluoride ion concentration, Ni ion leaching and cell efficiency.

## Figures and Tables

**Table 1:** Electrochemical parameters extracted from the open circuit potential versus time (*E*_OCP_–*t*) curves and the potentiodynamic polarization curves.

Samples	*E_OCP_* (V)	*E*_corr_ (V)	*i*_corr_ (μA cm^−2^)
NiTi	0.095	0.082	1.13
Pure Ti	−0.023	−0.419	28.80
Pure Ni	−0.397	−0.011	79.28

**Table 2:** The estimated ratios of different chemical states on the surface of electrodes.

Samples	Ni 2p (%)		Ti 2p (%)		O^2−^/OH^−^
	NiO + Ni(OH)_2_	Metallic Ni	TiO_2_	Metallic Ti	
NiTi	23.66	76.34	87.72	12.28	0.64
Pure Ti	/	/	88.5	11.5	2.94
Pure Ni	86.99	13.01	/	/	0.1

**Table 3:** Electrochemical parameters obtained from the equivalent circuits simulation (the EIS data fitting results before and after potentiostatic polarization).

Before potentiostatic polarization	**Elements**	**CPE_1_-Y_0_** **(Ω^−1^ cm^−2^ s^n^)**	**R_1_** **(Ω cm^2^)**	**CPE_2_-Y_0_** **(Ω^−1^ cm^−2^ s^n^)**	**R_2_** **(Ω cm^2^)**	**∑χ^2^**
	NiTi	7.19 × 10^−5^	4.27	4.14 × 10^−5^	3.25 × 10^4^	1.08 × −10^3^
	Pure Ti	8.49 × 10^−5^	2.71	9.06 × 10^−5^	3.42 × 10^4^	1.02 × −10^3^
	Pure Ni	5.34 × 10^−5^	8.54	2.31 × 10^−5^	219.20	2.16 × −10^3^
After potentiostatic polarization	**Elements**	**CPE_1_-Y_0_** **(Ω^−1^ cm^−2^ s^n^)**	**R_1_** **(Ω cm^2^)**	**CPE_2_-Y_0_** **(Ω^−1^ cm^−2^ s^n^)**	**R_2_** **(Ω cm^2^)**	**∑χ^2^**
	NiTi	4.59 × 10^−5^	5.64	4.54 × 10^−5^	4.14 × 10^4^	1.56 × −10^3^
	Pure Ti	2.65 × 10^−4^	6.97	1.35× 10^−4^	8.15 × 10^4^	1.57 × −10^3^
	Pure Ni	1.04 × 10^−4^	2.87	1.18 × 10^−4^	184.95	1.97 × −10^3^

## Data Availability

The data presented in this study are available on request from the corresponding author.

## Supplementary Materials

The following supporting information can be downloaded at https://www.mdpi.com/article/10.3390/molecules29153696/s1, Figure S1: The (a) scanning electron microcopy (SEM) image and (b) X-ray diffraction (XRD) pattern of the NiTi alloy prepared by conventional melting; Figure S2: Equivalent electrical circuit used for modeling experimental electrochemical impedance spectroscopy (EIS) data.
