# Supplementary material for: Corrosion and Interfacial Contact Resistance of NiTi Alloy as a Promising Bipolar Plate for PEMFC
Source: Molecules. 2024 Aug 5;29(15):3696. doi: 10.3390/molecules29153696 (PMC11313899; doi:10.3390/molecules29153696)
Supplement: Supplementary file 1 [file molecules-29-03696-s001.zip › molecules-3064499-supplementary.pdf]

Supplementary Materials for

Corrosion and Interfacial Contact Resistance of NiTi Alloy as a Promising Bipolar Plate for PEMFC

Yingping Li [1,†], Xiaofen Wang [2,3,†], Yuanyuan Li [1,2,4,*], Zhuo He [2], Guohong Zhang [4], Zhen Wang [4], Shaohua Wang [5,*], Fei Hu [2] and Qiongyu Zhou [2]

[1] The State Key Laboratory of Refractories and Metallurgy, Wuhan University of Science and Technology, Wuhan 430081, China; liyingping@wust.edu.cn
[2] Key Laboratory of Green Surface Technology and Functional Coatings for Materials, China National Light Industry, Foshan University, Foshan 528000, China; wangxiaofen@shu.edu.cn (X.W.); 20220580212@stu.fosu.edu.cn (Z.H.); mfhufei@126.com (F.H.); zhouzhouqiongyu@fosu.edu.cn (Q.Z.)
[3] School of Materials Science and Engineering, Shanghai University, Shanghai 200444, China
[4] Analytical and Testing Center, Wuhan University of Science and Technology, Wuhan 430081, China; zhangguohong@wust.edu.cn (G.Z.); wangzhen@wust.edu.cn (Z.W.)
[5] School of Materials Science and Engineering, Jingdezhen Ceramic University, Jingdezhen 333403, China
* Correspondence: liyuanyuan@wust.edu.cn (Y.L.); wsh-501@163.com (S.W.); Tel.: +86-13512741496 (ext. 430081) (Y.L.); +86-18379792280 (ext. 333403) (S.W.)
† These authors contributed equally to this work.

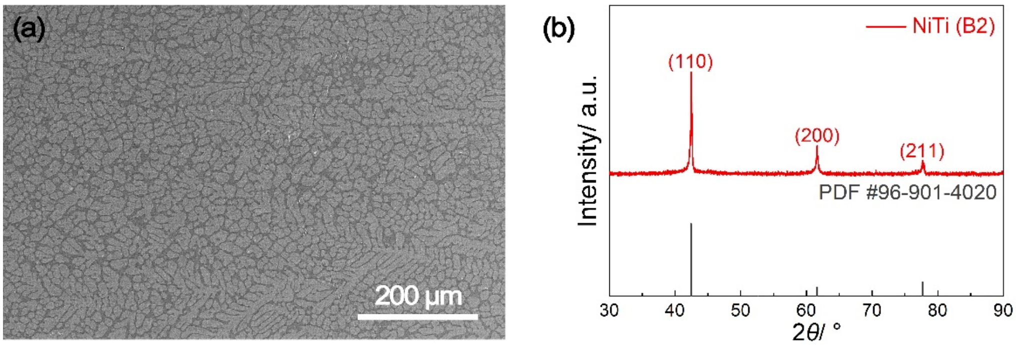

Figure S1. The (a) scanning electron microcopy (SEM) image and (b) X-ray diffraction (XRD) pattern of the NiTi alloy prepared by conventional melting

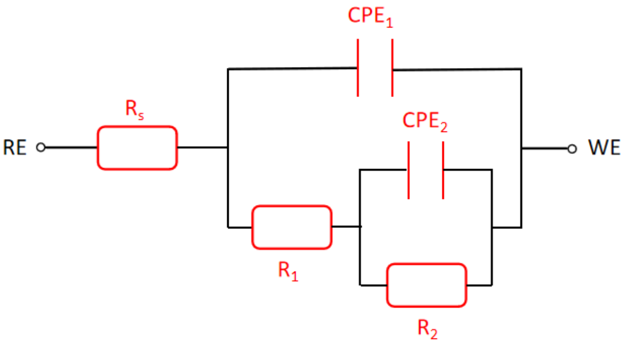

Figure S2. Equivalent electrical circuit used for modeling experimental electrochemical impedance spectroscopy (EIS) data
